# The Potential of Systems Biology to Discover Antibacterial Mechanisms of Plant Phenolics

**DOI:** 10.3389/fmicb.2017.00422

**Published:** 2017-03-16

**Authors:** Caroline S. Rempe, Kellie P. Burris, Scott C. Lenaghan, C. Neal Stewart

**Affiliations:** ^1^College of Arts and Sciences, Graduate School of Genome Science and Technology, University of TennesseeKnoxville, TN, USA; ^2^Department of Food Science, University of TennesseeKnoxville, TN, USA; ^3^Department of Food, Bioprocessing and Nutrition Sciences, North Carolina State UniversityRaleigh, NC, USA; ^4^Department of Mechanical, Aerospace, and Biomedical Engineering, University of TennesseeKnoxville, TN, USA; ^5^Department of Plant Sciences, University of TennesseeKnoxville, TN, USA

**Keywords:** antimicrobials, mechanism of action, membrane, metabolic processes, microbial structure

## Abstract

Drug resistance of bacterial pathogens is a growing problem that can be addressed through the discovery of compounds with novel mechanisms of antibacterial activity. Natural products, including plant phenolic compounds, are one source of diverse chemical structures that could inhibit bacteria through novel mechanisms. However, evaluating novel antibacterial mechanisms of action can be difficult and is uncommon in assessments of plant phenolic compounds. With systems biology approaches, though, antibacterial mechanisms can be assessed without the bias of target-directed bioassays to enable the discovery of novel mechanism(s) of action against drug resistant microorganisms. This review article summarizes the current knowledge of antibacterial mechanisms of action of plant phenolic compounds and discusses relevant methodology.

## Introduction

Each group of multi-drug resistant bacteria has been directly linked to thousands of deaths annually across the globe, with resistant *Shigella* species and *Mycobacterium tuberculosis* each causing more than one million deaths annually (WHO, 2014). In the United States (U.S.), drug resistant bacteria are the primary cause of more than 23,000 deaths and 2 million serious infections each year, of which resistant *Clostridium difficile, Enterobacteriaceae* species, and *Neisseria gonorrhoeae* are considered “urgent threats” (CDC, 2013). These numbers will continue to rise as multi-drug resistant bacteria become more prevalent; the recent documentation of colistin-resistant *Escherichia coli* in the U.S. suggests that even “last resort” antibiotics with major side effects are likely to lose their effectiveness (McGann et al., 2016).

Natural products, including compounds obtained from plants, have had renewed attention for their diverse structures and bioactive characteristics. Phenolic compounds found in plants can be used to combat multi-drug resistant bacteria (reviewed by Abreu et al., 2012), but their mechanisms of action must be thoroughly characterized before they can be rationally used as antibacterial treatments.

The general structural categories of plant-derived phenolics, as categorized by Cowan (1999), include simple phenolics, phenolic acids, quinones, flavonoids/flavones/flavonols, coumarins, and tannins. Although phenolics are classified as compounds with a hydroxylated aromatic ring, Cowan categorized flavones as “phenolic structures containing one carbonyl group” (Cowan, 1999), which prompted the inclusion of flavones in this work. A useful review of phytochemical classes and their general antibacterial modes of action has already been compiled (Borges et al., 2015), as has as a detailed review of flavonoid mechanisms of action, including cytoplasmic membrane damage, topoisomerase inhibition, NADH-cytochrome c reductase inhibition, and ATP synthase inhibition (Cushnie and Lamb, 2011). More recent flavonoid reviews have focused on mechanisms of action involving cell membranes (Tsuchiya, 2015; Verstraeten et al., 2015). The mechanisms of action of phenolic compounds were additionally reviewed with an emphasis on structural features correlated to specific mechanisms (Gyawali and Ibrahim, 2014).

Most methods to determine antibacterial mechanisms are target-directed assays that directly test single proteins or other cellular targets *in vitro*. Target-directed assays are very informative since established bioassay protocols against a single target can definitively assign binding and inhibitory activity of compounds with enzymes known to be essential to cell survival. However, target-directed assays also limit the scope of mechanisms that can be identified, which is undesirable when existing antibacterial mechanisms of action threaten to become obsolete with the rise of resistant bacteria. This is where undirected approaches like systems biology have value, in the broad, unbiased survey of possible mechanisms that can be used to formulate new hypotheses for novel target-directed assays.

In this review, we aim to assess the current state of mechanistic investigations of plant phenolic antibacterial compounds and survey systems biology approaches that have demonstrated success or potential for identifying antibacterial mechanisms.

## Membrane disruption by phenolic compounds

Membrane disruption, in both Gram-positive and Gram-negative bacteria, contributes to the antibacterial activity of most plant phenolics that have been mechanistically assessed (Supplementary Table 1; abbreviated in Table 1). Many of the membrane integrity analyses cited in Supplementary Table 1 combined several common, simple methods to monitor the influx of hydrophobic dyes or antibiotics, the efflux of intracellular constituents, and microscopic observation. While these membrane permeability analyses provide valuable information about membrane disruption, they give little, if any, direction to the specific major mechanisms of action of a compound. Furthermore, these analyses generally leave an open question as to whether the compound altered membrane stability by interfering with intracellular processes or by direct interaction with membrane components. Other more unique assays have also been conducted, for example, in the quantification of cell surface hydrophobicity the tendency of a hydrophilic water drop to spread or bead when in contact with a bacterial lawn was measured (Borges et al., 2013). This assay more specifically characterized the effect of gallic and ferulic acids, which were observed to increase surface hydrophilicity of Gram-negative bacteria and hydrophobicity of Gram-positive bacteria (Borges et al., 2013). Nevertheless, this measure of surface hydrophobicity could also be attributed to either direct interaction with the membrane or an interaction with intracellular components that impact cell wall chemistry.

**Table 1:** **Known antibacterial mechanisms of action of phenolic compounds**.

**Structure**	**Phenolic**	**Organisms**	**Mechanisms**	**Methods**	**References**
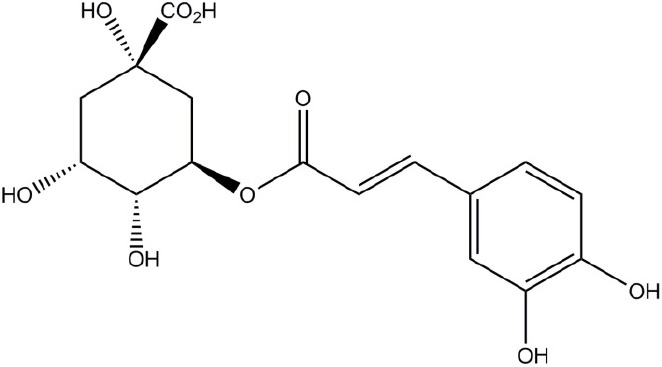	Chlorogenic acid	*Streptococcus pneumoniae, Staphylococcus aureus, Bacillus subtilis, Escherichia coli, Shigella dysenteriae, Salmonella Typhimurium*	Cell membrane disruption	Efflux of cell components, uptake of hydrophobic antbiotics, intracellular pH, membrane potential, microscopy	Lou et al., 2011; Li et al., 2014
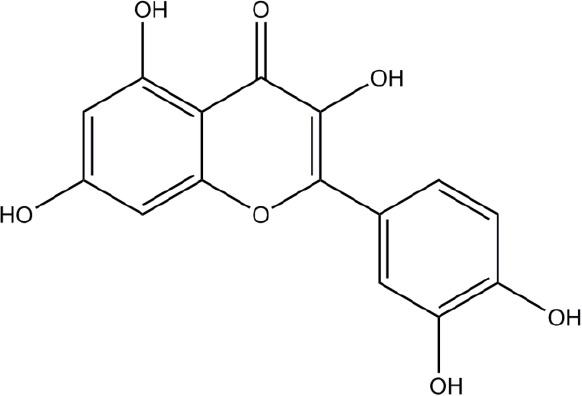	Quercetin	Model membrane, *Escherichia coli, Helicobacter pylori*	Cell membrane disruption (membrane rigidification), DNA intercalation, DNA gyrase inhibition, Type III secretion inactivation, dehydratase inhibition (HpFabZ), protein kinase inhibition	DNA-gyrase supercoiling inhibition assay, type III secretion assay by monitoring fluorescence of Glu-CyFur dye, interaction of molecules with large unilamellar vesicles (fluorescence monitoring), kinase enzyme inhibition assays	Plaper et al., 2003; Zhang et al., 2008; Shakya et al., 2011; Wu et al., 2013a,b; Tsou et al., 2016
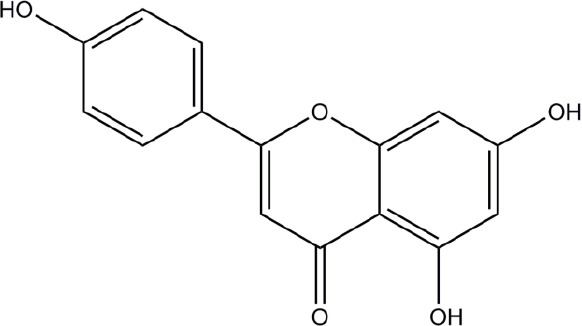	Apigenin	*Helicobacter pylori*	Dehydratase inhibition (HpFabZ), protein kinase inhibition	HpFabZ enzyme inhibition assay, interaction of molecules with large unilamellar vesicles (fluorescence monitoring), kinase enzyme inhibition assays	Zhang et al., 2008; Shakya et al., 2011

*MRSA, methicillin resistant Staphylococcus aureus; HRGC: high resolution gas chromatography*.

Once a membrane effect is known to exist, specific binding assays (Nakayama et al., 2015) or model membrane insertion assays (Wu et al., 2013a) can confidently test direct interactions between specific components. Model membranes have already been used to test the direct interaction of epicatechin gallate and epigallocatechin gallate with membranes (Hashimoto et al., 1999; Kumazawa et al., 2004). However, the extensive diversity in bacterial membrane composition (Sohlenkamp and Geiger, 2016) would likely require specific assays against each organism of interest. Another unique assay that may be useful in assessing direct membrane binding is a basic membrane integrity assay with exogenous magnesium added. This assessment revealed a dependence on magnesium akin to an ethylenediaminetetraacetic acid (EDTA) control for the membrane-disrupting activity of gallic acid (Nohynek et al., 2006). Since magnesium ions are known to stabilize Gram-negative bacterial membranes, this simple assay might easily test direct membrane interactions involving magnesium, though other integral cell functions involving magnesium should also be considered.

A particularly in-depth study was conducted for epigallocatechin gallate's mechanism of action against methicillin-resistant *Staphylococcus aureus* (MRSA) and its contribution to renewed beta-lactam susceptibility. The study assessed purified cell wall components, phosphorus content, the effects of exogenous peptidoglycan on activity, adherence to glass, penicillin binding protein abundance, Triton-X-100 autolysis, bacteriolytic enzymes, free lipoteichoic acid, and lysostaphin's pentaglycine cleavage activity. Each of these methods focused on addressing a particular hypothesis of epigallocatechin gallate's mechanism of action, which is an ideal, albeit time-consuming, approach for a mechanistic assessment. Nevertheless, the specific mechanism of action remained unknown despite the new knowledge of epigallocatechin gallate's observed effects on cell adherence, autolysin accumulation, and lipoteichoic acid release (Stapleton et al., 2007). Another method that has given mechanistic insight, but not a specific mechanism of action, was the use of high resolution gas chromatography (HRGC) lipid profiling to identify specific lipids altered by the presence of eugenol, carvacrol, or thymol in both Gram-negative and Gram-positive bacteria (Di Pasqua et al., 2007). All three tested phenolics were observed to increase major fatty acids (palmitic, oleic, cis-10 heptadecenoic acids) in Gram-negative bacteria while Gram-positive *S. aureus* lipid profiles changed across several fatty acids. *S. aureus* treatment with thymol, though, resulted in a clear increase in saturated fatty acids and decrease in unsaturated fatty acids, which could be due to a desaturase response to cell leakage (Di Pasqua et al., 2007). While this type of untargeted assessment requires interpretation, it gives a greater depth of information than most of the basic membrane disruption assays. When iterations of logically planned assays are performed, a good depth of information is also obtained. However, conducting iterations of assays is time and labor intensive while HRGC can gather information on many potential targets simultaneously. Although no liquid chromatography-mass spectrometry (LC-MS) lipidomics experiments to deduce the mechanisms of action of a phenolic were found in the literature, a basic LC-MS experiment can measure an even larger range of metabolites than HRGC and more confidently identify them; this technology can profile the relative abundance of thousands of lipids simultaneously and is easily automated (Wenk, 2005). Using more untargeted approaches capable of profiling thousands of compounds in a single experiment will be useful in the rapid generation of mechanistic hypotheses that can subsequently be tested with a relevant subset of targeted assays.

## Non-membrane mechanisms of action

Assessments for non-membrane mechanisms of action can also be categorized into target-directed assays for known mechanisms of action and undirected experiments that take a systems-wide approach to exploring mechanisms of action. Among the plant phenolics tested in Supplementary Table 1, targeted assays have discovered DNA gyrase inhibitory activity for 11 phenolic compounds (Ohemeng et al., 1993; Wu et al., 2013b), type III secretion inhibition for 9 compounds (Tsou et al., 2016), inhibition of helicase activity for 5 phenolic compounds (Xu et al., 2001), multi-drug efflux pump inhibitors for 3 compounds (Smith et al., 2007; Fiamegos et al., 2011; Bag and Chattopadhyay, 2014), dehydratase inhibition for 3 compounds (Zhang et al., 2008), protein kinase inhibition for 2 compounds (Shakya et al., 2011), and single phenolic compounds among which one inhibited urease activity (Moon et al., 2013), one bound iron (Chung et al., 1998), one inhibited succinate dehydrogenase and malate dehydrogenase (Yao et al., 2012), one intercalated into DNA (Lou et al., 2012), one induced DNA fragmentation, an ROS response, and suppressed FtsZ expression (Hwang and Lim, 2015), and one bound to the FtsZ protein and inhibited FtsZ assembly (Rai et al., 2008). Many of these results clearly come from large experimental activity screens in which several phenolic compounds were assessed for a single mechanism of action. This single mechanism screening approach generates valuable information and more such screens are needed to assess phenolic antibacterial mechanisms. Nevertheless, the major mechanism(s) of action of a compound could easily be overlooked with a screening approach if confounding multiple mechanisms exist for the target compound. This problem seems likely when looking at one of the most-studied phenolics, quercetin, and its diverse list of mechanisms of action [cell membrane disruption, DNA intercalation, DNA gyrase inhibition, type III secretion inactivation, dehydratase inhibition (HpFabZ), and protein kinase inhibition; Table 1].

A general methodological approach to these targeted assessments was to verify antibacterial activity, purify the target enzyme, and characterize its phenolic-bound and unbound structures using spectrophotometric techniques. As an example, after gathering antibacterial activity data and purifying the FtsZ enzyme, curcumin was observed to inhibit the FtsZ protofilament assembly by monitoring assembly kinetics with light scattering, to bind to purified FtsZ based on an increase in bound-curcumin fluorescence at 495 nm, and was noted to alter the secondary structure of FtsZ based on a circular dichroism analysis (Rai et al., 2008). In some studies, however, only inhibitory activity was determined and structural binding characteristics were not assessed (Ohemeng et al., 1993; Smith et al., 2007; Bag and Chattopadhyay, 2014). The most specific interaction details were found by the two studies that obtained crystal structures of quercetin or apigenin bound to the enzymes they inhibited (Zhang et al., 2008; Shakya et al., 2011). One of these studies moved to the next step in mechanistic understanding by introducing a single amino acid mutation to the enzyme in order to verify the importance of a key binding interaction (Zhang et al., 2008). Unfortunately, the conserved tyrosine that was mutated did not alter binding, so the specific, necessary interaction points between quercetin or apigenin and FabZ still need to be validated (Zhang et al., 2008). Two additional studies were performed in which detailed structure mechanisms were determined with molecular docking (Plaper et al., 2003; Fiamegos et al., 2011). Detailed structural characterizations of phenolic binding mechanisms are a valuable contribution to the field that can facilitate successful computational prioritizations of potentially active compounds and predictions of compound activities to guide both natural product testing and rational antibacterial design.

Only one study in Supplementary Table 1 that is not membrane-specific can be called untargeted: the proteomics assessment of thymol antibacterial activity against *Salmonella* Thompson (Di Pasqua et al., 2010). Researchers found altered abundances of citric acid cycle enzymes, ATP synthesis enzymes, stress-related chaperone proteins, cell envelope proteins, and an absence of the antioxidant protein thioredoxin 1 in treated cells. Although a specific mechanism of action was not identified here, valuable information was produced that verified the cell membrane disruption observed in other thymol-treated Gram-negative bacteria and added several additional possible protein targets. Additional assays to test different portions of the impacted pathways, thioredoxin, and relevant regulatory proteins are still needed, of course, but it is now clear that membrane disruption alone was not the sole mechanism of action of thymol against *S*. Thompson and that intracellular proteins, including thioredoxin, may be important targets (Di Pasqua et al., 2010).

## Key functional characteristics

**Figure 1:**
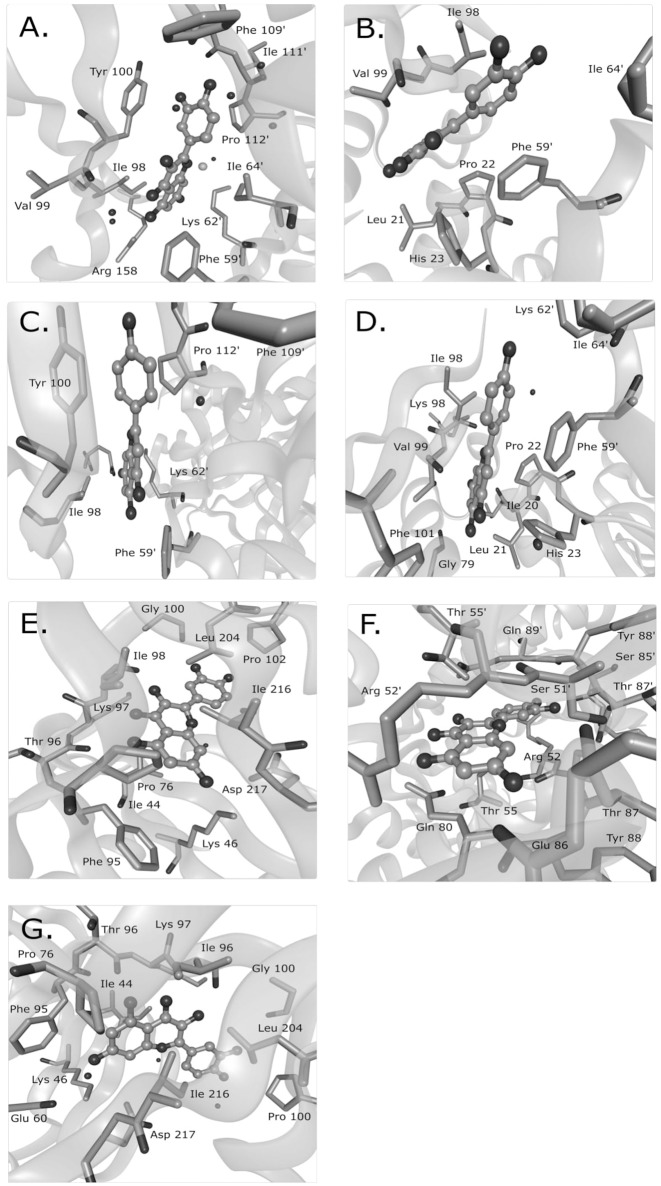
**Known binding sites of phenolic compounds and their antibacterial targets from crystal structure data**. Quercetin was observed to bind to the *Helicobacter pylori* beta-hydroxyacyl-acyl carrier protein (HpFabZ) at two sites (**A,B**; PDB structure 3CF8), apigenin bound to HpFabZ at the same two sites (**C,D**; PDB structure 3CF9), and quercetin bound to the kinase APH(2″)-IVa in three locations [**E,F,G**; PDB structure 4DFU (formerly 3R82)]. Isolated black spheres represent water and isolated gray spheres represent chloride ions. The ligand is in ball-and-stick representation while the protein side chains in contact range are in stick representation. PDB, protein data bank. This figure was made with BALLView (Moll et al., 2006).

Mechanisms characterized to the level of atomic interaction points are rare in the examples of Supplementary Table 1, but give the most specific mechanistic information. Crystal structures of FabZ with quercetin or apigenin as ligands revealed that both ligands similarly blocked substrates from passing through a tunnel to reach the FabZ active site (Zhang et al., 2008). Hydrophobic interactions likely sandwiched the phenolic ring of quercetin or apigenin between a tyrosine and proline in one binding site and between a phenylalanine and an isoleucine in a second binding site (Figure 1). In the crystal structure of quercetin-bound resistance kinases, hydrogen bonds between hydroxyl groups, and nearby amino acids appeared to be the primary interactions (Shakya et al., 2011; Figure 1). Based on several flavonoids tested, the absence of a particular carbonyl group was necessary for binding and the hydroxyl position was important (Shakya et al., 2011). In a molecular docking study of quercetin and DNA gyrase, it was proposed that quercetin has one mechanism by which it binds DNA, stabilizes the DNA-gyrase complex, and induces DNA cleavage in addition to a second mechanism by which it inhibits DNA supercoiling by competitively binding to the ATP binding site of the DNA gyrase B subunit (Plaper et al., 2003). Another molecular docking study surveyed the localization of caffeoylquinic acids to an efflux pump and determined that caffeoylquinic acids tended to bind in a position that blocked the efflux pump (Fiamegos et al., 2011).

Similar trends in functional groups that contribute to bioactivity are seen in many structure-activity relationship studies. Electron distribution, as impacted by the number and location of hydroxyl groups and double bonds, has been the major factor associated with antibacterial activity of flavonoids (reviewed by Shapiro and Guggenheim, 1998; Wu et al., 2013a,b; Gyawali and Ibrahim, 2014). Electron localization and other structural features also impact molecular hydrophobicity, which impacts the types of membrane interactions that are possible.

Other studies have found hydroxyl counts and positions to be important for bioactivity extending beyond flavonoids and into the broader category of phenolics. A quantitative structure activity relationship (QSAR) analysis with more than 100 phenolics and related compounds used MIC doses for the oral bacteria *Porphyomonas gingivalis, Selenomonas artemidis*, and *Streptococcus sobrinus* and found that a hydroxyl group attached to an aromatic ring was required for low MICs (Shapiro and Guggenheim, 1998). Exceptions to the general trend of key hydroxyl placement have been observed through isomers that had different antibacterial activities, suggesting that chirality is also an important factor (Friedman et al., 2002; reviewed by Gyawali and Ibrahim, 2014). Interestingly, the number of hydroxyl groups has also been correlated with antioxidant activity (reviewed by Rice-Evans et al., 1996; Balasundram et al., 2006). However, any relationship between antioxidant activity and antibacterial activity is not fully understood.

## Synergistic antibacterial activity

If specific mechanisms of action are known, phenolic compounds can be rationally combined with other antibacterial compounds to synergistically combat multi-drug resistant bacteria. Synergistic effects have already been observed for many phenolic compounds when combined with antibiotics currently in use (reviewed by Aiyegoro and Okoh, 2009; Hemaiswarya and Doble, 2010; Amin et al., 2015; essential oil components reviewed by Langeveld et al., 2014; Oh and Jeon, 2015; Lim et al., 2016). Many synergistic effects have been attributed to membrane-disrupting compounds, including phenolics, which allow intracellular toxins faster/easier access to their targets (Hemaiswarya and Doble, 2010; Amin et al., 2015; Oh and Jeon, 2015). Additionally, strains that use efflux pumps to remove toxins can be attacked by blocking the efflux pumps with one compound and applying an intracellular toxin as a second compound for synergistic killing (Oh and Jeon, 2015; reviewed by Tegos and Stermitz, 2002; Prasch and Bucar, 2015). The down-regulation of efflux pump expression has also been observed (Oh and Jeon, 2015). Synergistic effects between other modes of action may also exist and could improve the functionality of existing antibacterial compounds, especially in combating drug resistance mechanisms.

## Mammalian toxicity

Many phenolic compounds have been tested for their cytotoxicity against different cancer cell lines, but limited information is available for effects on non-tumorigenic cells or whole organisms. Vanillin is one compound with a clear toxicity rating in the literature; it is considered non-toxic at typical exposure concentrations with an LD_50_ of 3500–4000 mg/kg in acute toxicity tests in rats based on assays summarized by the Organization for Economic Co-operation and Development (OECD). *In vivo* studies in mice showed no genotoxicity and vanillin was actually observed to decrease the mutagenic effect of the positive controls mitomycin C and ethylnitrosourea in mouse micronucleus assays (OECD SIDS, 1996). Other phenolic compounds have been extensively reviewed for their toxic properties by Galati and O'Brien (2004), who highlighted the pro-oxidant effects of compounds in the presence of metals and peroxidases, DNA binding of compounds with catechol groups, and mouse hepatotoxicity of epicatechin gallate and propyl gallate. While most compounds derived from edible plants, including phenolics, are considered safe at common levels of consumption, rigorous toxicity testing must be done to ensure safety at different concentrations and in different conditions.

## Systems biology methods

**Figure 2:**
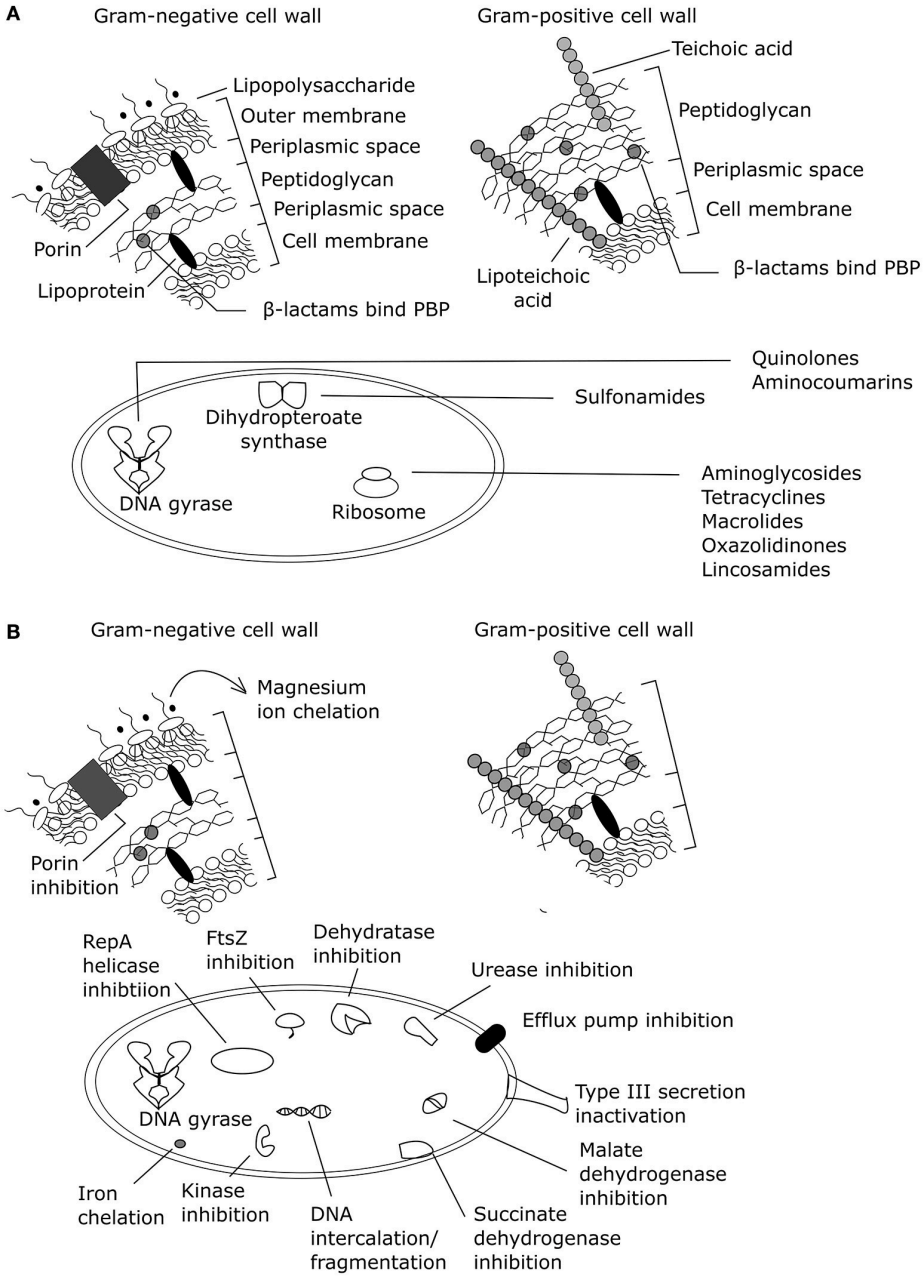
**Antibacterial mechanisms of action summarized for (A)** common antibiotic classes and **(B)** plant phenolic compounds (adapted from Helander et al., 1997; Kohanski et al., 2010; Brown et al., 2015). PBP, penicillin binding protein. Effects of exogenous magnesium were not tested on a Gram-positive organism, only a Gram-negative organism.

Phenolic compounds from natural sources have been assessed for antibacterial mechanisms related to membranes and specific protein and/or pathway targets (Supplementary Table 1; Figure 2). However, most of these assays are limited to target-directed tests of known antibacterial mechanisms and do not facilitate the discovery of novel mechanisms of action or multiple mechanisms of action. Increasing the use of undirected systems biology approaches (summarized in Table 2) could reveal the major known and/or unknown mechanism(s) of action.

**Table 2:** **Summary of systems biology methods for the determination of antibacterial mechanism of action**.

**Method**		**Information obtained**	**Advantages**	**Limitations**
Chemical-chemical screen		A “fingerprint” of synergy interactions between the chemical of interest and compounds with known antimicrobial activity	Can develop hypotheses about mechanisms of action	Limited to hypotheses based on known mechanisms of action, pairwise synergy tests may be large and time-consuming
Chemical-genetic screen		A “fingerprint” of activity profiles of a set of deletion and overexpression mutants	Can develop hypotheses about mechanisms of action	Limited to mechanisms related to mutants
Proteomics	Affinity chromatography	Identifies interactions between a tagged antimicrobial compound and any protein	Definitive evidence of protein-ligand interactions	Requires strong protein-ligand affinity, misses low-abundant proteins, requires ligand tag that does not inactivate antimicrobial activity
	Phage display	Identifies interactions with phage-expressed proteins	Definitive evidence of protein-ligand interactions, can capture low-abundance proteins	Eukaryotic proteins may be mistranslated or misfolded, may be non-specific binding, not good for multimeric or transmembrane proteins
	Microarray	Identifies interactions with purified proteins attached to a slide	Definitive evidence of protein-ligand interactions	Difficult to purify many proteins for a protein microarray
	Expression analysis	All proteins altered by the presence of an antimicrobial are observed	Patterns in expressed proteins can reveal specific antimicrobial mechanisms	Data interpretation can be difficult
Transcriptomics	Microarray	Survey of expression altered by antimicrobial compound	Patterns in transcribed RNA can reveal specific antimicrobial mechanisms	Limited to known transcripts, data interpretation can be difficult
	RNA-seq	All transcripts altered by the presence of an antimicrobial are observed	Patterns in transcribed RNA can reveal specific antimicrobial mechanisms	Data interpretation can be difficult
Metabolomics		All metabolites altered by the presence of an antimicrobial are observed	Patterns in metabolites can reveal specific antimicrobial mechanisms	Data interpretation can be difficult
Genomics of screened mutants		Genetic mutant bacteria with resistance to the tested antimicrobial are sequenced to identify the mutation	Mutations found in the genome can give direct evidence of mechanism of action	Mutations may merely reveal a generic resistance response (i.e., multi-drug efflux pump activity)
Screening for targets		Screen for possible antimicrobial targets, then use target-directed screens to evaluate targets	Identifies putative targets	Limited by target selection criteria, limited by diversity of chemical structures in the second step of target-directed screening, has had very minimal success in the past
Structural systems pharmacology		Acquire data from multiple-omics technologies, develop hypotheses/models of system	Integrating multiple types of data can give more specific and conclusive evidence of mechanisms of action	Data interpretation can be difficult

A small set of systems biology approaches are limited to assessing known antibacterial mechanisms and can thus be considered target-directed. These include chemical-genetic and chemical-chemical interaction approaches. Chemical-genetic approaches collect a genetic “fingerprint” from the effect of antibiotic treatment on a collection of overexpression and deletion mutants that can be used to generate hypotheses about mechanism of action. Similarly, chemical-chemical interaction analyses collect a “fingerprint” of synergistic data using pairwise combinations of the unknown chemical and chemicals with known mechanisms. These methods have had success, as reviewed in Farha and Brown (2016), but are limited to the characterized mutants or chemicals of known mechanism that are used in the assessments.

Other systems biology approaches are diverse, but use an undirected approach to survey sets of biomolecules that allows for the discovery of novel antibacterial targets. Proteomics approaches have been particularly popular for the discovery of small molecule binding targets since definitive evidence of protein-ligand binding can be obtained. Affinity chromatography, phage display of peptides, and protein microarrays have been used to effectively isolate binding targets of small molecules for separation and subsequent identification with mass spectrometry. These proteomic techniques have already been thoroughly reviewed (Wong et al., 2008; Ziegler et al., 2013). Expression proteomics can also be effective in identifying mechanisms of action based on identifying proteins differentially expressed with an antibacterial treatment. Recently, expression proteomics showed its usefulness in mechanistic assessments by providing evidence for a second mechanism of action of the atypical tetracycline chelocardin, which inhibits peptidyl transferase at low concentrations and causes membrane depolarization at high concentrations in *B. subtilis* (Stepanek et al., 2016).

Metabolomics has also proven useful for determining mechanisms of action that impact specific metabolic functions. The mechanisms of action of a thymidine kinase inhibitor (AZ1), a 1-deoxy-D-xylulose-5-phosphate (DXR) pathway inhibitor (fosmidomycin), and a lipid A synthesis inhibitor (CHIR-090) were readily identified from *E. coli* metabolomics data by researchers blinded to the antibiotics used (Vincent et al., 2016). However, in the analysis of other compounds, the metabolomics data showed non-specific upregulation of many metabolites for a peptidoglycan cross-linking inhibitor, a DNA ligase inhibitor, and an enoyl-acyl carrier protein inhibitor. Furthermore, no discernible metabolic differences were observed between a control and an antibiotic treatment that uncoupled the proton gradient of the electron transport chain. Measurement limitations in observing peptidoglycan molecules (too large) and mono-, di-, and tri-phosphates (differing ionizabilities) were possible reasons for the lack of relevant information from the peptidoglycan inhibitor and oxidative phosphorylation inhibitor (Vincent et al., 2016). Researchers were also able to differentiate antibacterial compounds based on the affected *E. coli* metabolome after 60 and 90 min of treatment, but not after 30 min (Belenky et al., 2015). It was not specified whether the known cellular targets of ampicillin, kanamycin, and norfloxacin could be determined from their respective metabolomes, but the ROS hypothesis of cell death, which theorizes that ROSs induce cell death in all antibacterial mechanisms of action, was supported based on similarly high levels of a metabolic marker for DNA/RNA oxidation across all antibiotic treatments (Belenky et al., 2015). Another metabolomic study successfully used the exometabolome of *S. aureus* to determine the previously unknown antibacterial mechanism of action of triphenylbismuthdichloride (TPBC). By comparing the exometabolome of TPBC-treated *S. aureus* to the exometabolomes of kanamycin, ciprofloxacin, trimethoprim, and fluoropyruvate it was observed that only TPBC and fluoropyruvate treatments resulted in continuously accumulated pyruvate. Thus, it was hypothesized that TPBC had pyruvate dehydrogenase inhibition activity, which was verified by an enzyme activity assay with cell lysate (Birkenstock et al., 2012). Another study used a similar comparison methodology to hypothesize the mechanism of the non-phenolic plant natural product dihydrocucurbitacin F-25-O-acetate. The metabolic profile of *S. aureus* treated with dihydrocucurbitacin F-25-O-acetate clustered with the metabolic profile of *S. aureus* treated with vancomycin, a known inhibitor of peptidoglycan synthesis (Biao-Yi et al., 2008).

A scheme to classify compounds into known mechanisms of action was also recently used with NMR metabolomics, where a partial least squares discriminant analysis of the metabolic “fingerprints” of *E. coli* treated with one of nine antibiotics of known mechanism gave 91% accuracy with intracellular data and 30% accuracy with extracellular data (Hoerr et al., 2016). Another systems biology approach similarly classified phenotypes based on the Raman spectra of dried *E. coli* cells to distinguish antibacterial mechanisms of action; 15 antibacterial compounds representing protein synthesis inhibitors, cell wall synthesis inhibitors, DNA synthesis inhibitors, and RNA synthesis inhibitors were differentiable with 83.6% accuracy by mechanistic class. Ampicillin was always incorrectly classified with the final model based on a linear discriminant analysis of principle components, but overlap between the mechanism groups could explain this and other misclassification tendencies (Athamneh et al., 2014). Classification schemes like these can only classify data into known mechanisms of action. However, such best fit classifications can potentially be of value by singling out unknown mechanisms of action that do not group into known mechanistic classes.

Another systems approach is the high-throughput but still time intensive method of screening for novel targets by, e.g., using genomes to identify genes that are conserved in multiple bacterial organisms followed by *in vitro* enzyme assays to screen for inhibitors of purified targets (discussed by Miesel et al., 2003; Payne et al., 2007; Harvey et al., 2015). This approach was widely adopted by industry when basic screens for antibacterial activity began to reveal the same set of common antibacterial compounds. However, it was surprisingly unsuccessful at identifying novel compounds, possibly due to poor selection of targets and chemical diversity in the libraries used to screen selected targets (discussed by Payne et al., 2007; Harvey et al., 2015).

Transcriptomic studies have tended to highlight pathways impacted by an antibacterial compound without identifying a specific mechanism of action (Yu et al., 2012; Elnakady et al., 2016), although transcriptional profiling of bacterial mutants revealed expression trends that were used to predict phenylalanyl-tRNA synthetase inhibition and bacterial acetyl coenzyme A carboxylase inhibition as the primary mechanisms of action of two novel antibacterial compounds (Freiberg et al., 2005). A 2004 review cites transcriptomic and proteomic studies that assessed antibacterial mechanisms, some of which were successful in identifying mechanisms of action (Freiberg et al., 2004).

Structural systems pharmacology has also made strides in identifying antibacterial mechanisms through combining multiple-omics approaches. A recent study was able to use an *E. coli* K12 genome scale metabolic model integrated with protein structures to correctly predict the antibacterial mechanisms of fosfomycin, and sulfathiazole. This model was also able to predict additional mechanisms of action for (1-hydroxyheptane-1,1-diyl)bis(phosphonic acid) and cholesteryl oleate and predict potential inhibitors of a protein target (tryptophan synthase beta subunit) with no previously known inhibitors. However, many false positives were also observed, which could be attributed to the static protein structures used to evaluate ligand binding (Chang et al., 2013).

The major limitations of -omics approaches are the ranges of detection for different technologies, the availability of annotated information for a given organism, and data interpretation challenges. As an example, metabolomics high resolution LC-MS systems can give a good representation of major bacterial metabolic pathways, but lipids, high molecular-weight compounds, and volatile compounds generally require separate runs on different instrument platforms (reviewed by Dettmer et al., 2007; Aretz and Meierhofer, 2016). On the annotation aspect, bacterial pathogens have long been a focal point of bacterial research, so they are relatively well-studied and have readily accessible, consistently annotated databases specifically dedicated to pathogens (Wattam et al., 2014), though annotated genomes, transcriptomes, proteomes, and metabolomes in general are still far from comprehensive (Médigue and Moszer, 2007; Aretz and Meierhofer, 2016). The large scale of data generated by -omics technologies can be challenging to analyze and may not be useful in antibacterial target discovery if generic widespread stress effects occur or if no visible effects are observed. However, successes have already been observed and the need for novel antibacterial approaches necessitates undirected approaches that allow for the discovery of novel mechanisms of action. Moving forward, proteomics, and metabolomics seem particularly promising since they have successfully contributed to several determinations of antibacterial mechanisms of action. As reference libraries of peptides and metabolites expand, we expect these –omics technologies to be integrated into antibacterial discovery pipelines to guide targeted assays and allow for the discovery of novel mechanisms of action.

## Conclusion

The mechanisms of action of most naturally derived phenolic compounds are not well-characterized. Of the compounds reviewed here, mechanistic assessments have found specific antibacterial targets, including DNA, DNA gyrase, multi-drug efflux pumps, FabZ, protein kinases, helicase, and FtsZ. The identification of these antibacterial targets through target-directed methods enables the rational use of phenolic compounds against bacterial pathogens susceptible to these known mechanisms of action. However, target-directed approaches have no opportunity to discover novel mechanisms of action, which is a necessary step to combat multi-drug resistant bacteria. A systems-biology approach to investigating the antibacterial mechanisms of phenolic compounds is not yet common in determining phenolic antibacterial mechanisms of action, but will likely push the field forward by speeding up mechanistic determinations and removing the bias of testing for currently known mechanisms.

## Author contributions

CR, KB, SL, and CS conceived, researched, wrote, and edited this review. All authors read and approved the final manuscript.

### Conflict of interest statement

The authors declare that the research was conducted in the absence of any commercial or financial relationships that could be construed as a potential conflict of interest.
