# Supplementary material for: The Potential of Systems Biology to Discover Antibacterial Mechanisms of Plant Phenolics
Source: Front Microbiol. 2017 Mar 16;8:422. doi: 10.3389/fmicb.2017.00422 (PMC5352675; doi:10.3389/fmicb.2017.00422)
Supplement: Supplementary file 1 [file Table1.PDF]

Structure	Phenolic	Organisms	Mechanisms	Methods	References
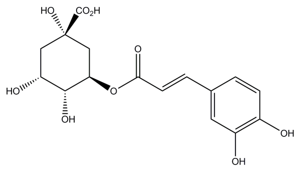	chlorogenic acid	*Streptococcus pneumoniae, Staphylococcus aureus, Bacillus subtilis, Escherichia coli, Shigella dysenteriae, Salmonella Typhimurium*	cell membrane disruption	efflux of cell components, uptake of hydrophobic antbiotics, intracellular pH, membrane potential, microscopy	Lou et al. 2011, Li et al. 2013
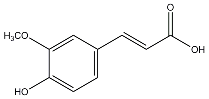	ferulic acid	*Pseudomonas aeruginosa, Escherichia coli, Staphylococcus aureus, Listeria monocytogenes, Cronobacter sakazakii*	cell membrane disruption	efflux of cellular components, uptake of hydrophobic dyes, intracellular pH and ATP concentration, membrane potential, surface zeta potential, membrane hydrophobicity using contact angles, microscopy	Borges et al. 2013, Shi et al. 2016
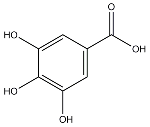	gallic acid	*Pseudomonas aeruginosa, Escherichia coli, Staphylococcus aureus, Listeria monocytogenes, Salmonella Typhimurium*	cell membrane disruption (Mg^{2+} chelation)	efflux of cellular components, uptake of hydrophobic dyes, intracellular pH and ATP concentration, membrane potential, surface zeta potential, membrane hydrophobicity using contact angles, exogenous magnesium added to membrane integrity assay	Borges et al. 2013, Nohynek et al. 2006

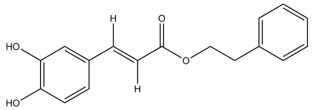	caffeic acid phenethyl ester	*Escherichia coli, Staphylococcus aureus*	cell membrane disruption)	efflux of cellular components, uptake of hydrophobic dye	Meyuhas et al. 2015
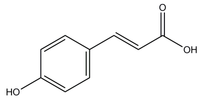	p-coumaric acid	*Streptococcus pneumoniae, Staphylococcus aureus, Bacillus subtilis, Escherichia coli, Shigella dysenteriae, Salmonella Typhimurium*	cell membrane disruption (outer membrane), DNA intercalation	efflux of cellular components, uptake of hydrophobic antbiotics, competetive binding of DNA with ethidium bromide	Lou et al. 2012
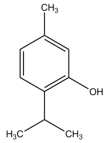	thymol	*Escherichia coli, Salmonella Typhimurium, Pseudomonas fluorescens, Staphylococcus aureus, Brochotrix thermosphacta, Salmonella Thompson*	cell membrane disruption (reduced unsaturated fatty acids), energy generation	efflux of cellular components, uptake of hydrophobic dyes, membrane polarization, intracellular pH, microscopy, HRGC	Xu et al. 2008, Lambert et al. 2001, Di Pasqua et al. 2007, Walsh et al. 2003, Helander et al. 1998, Di Pasqua et al. 2010
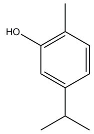	carvacrol	*Escherichia coli, Salmonella Typhimurium, Pseudomonas fluorescens, Staphylococcus aureus, Brochotrix thermosphacta, Bacillus cereus, Lactobacillus plantarum, Listeria innocua*	cell membrane disruption (reduced unsaturated fatty acids)	efflux of cellular components, uptake of hydrophobic dyes, membrane polarization, intracellular pH, membrane potential, microscopy, HRGC	Xu et al. 2008, Lambert et al. 2001, Di Pasqua et al. 2007, Fitzgerald et al. 2004, Ultee et al. 1999, Helander et al. 1998, Gill and Holley 2006

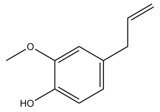	eugenol	*Salmonella Typhi, Escherichia coli, Salmonella Typhimurium, Pseudomonas fluorescens, Staphylococcus aureus, Listeria monocytogenes, Lactobacillus sakei*	cell membrane disruption (energy generation, reduced unsaturated fatty acids)	efflux of cellular components, uptake of hydrophobic dyes, intracellular pH, microscopy, HRGC	Devi et al 2010, Gill and Holley 2004, Walsh et al. 2003, Gill and Holley 2006, Di Pasqua et al. 2007
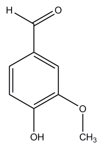	vanillin	*Escherichia coli, Lactobacillus plantarum, Listeria innocua*	cell membrane disruption	efflux of cellular components, membrane polarization, respiration	Fitzgerald et al. 2004
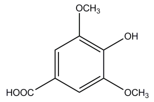	syringic acid	*Cronobacter sakazakii*	cell membrane disruption	uptake of hydrophobic dyes, membrane potential, microscopy	Shi et al. 2016
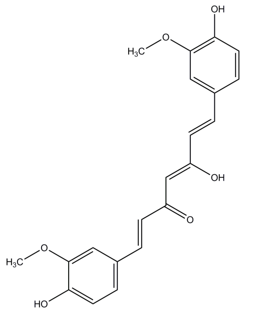	curcumin	*Escherichia coli, Salmonella Typhimurium, Photobacterium leiognathi, Bacillus subtilis, Staphylococcus aureus, Enterococcus faecalis, Pseudomonas aeruginosa*	cell membrane disruption, cell elongation (inhibited FtsZ protofilament assembly, FtsZ binding)	efflux of cellular components, uptake of hydrophobic dyes, membrane potential, microscopy, GTPase activity assay for FtsZ	Tyagi et al. 2015, Rai et al. 2008, Yun and Lee 2016
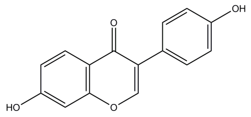	daidzein	model membrane, *Escherichia coli*	cell membrane disruption	interaction of molecules with large unilamellar vesicles (fluorescence monitoring)	Wu, He et al. 2013

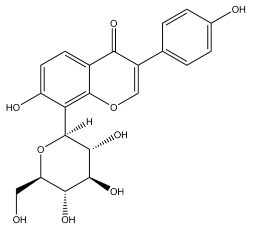	puerarin	model membrane, *Escherichia coli*	cell membrane disruption	interaction of molecules with large unilamellar vesicles (fluorescence monitoring)	Wu, He et al. 2013
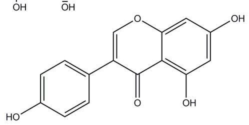	genistein	model membrane, *Escherichia coli*	cell membrane disruption	interaction of molecules with large unilamellar vesicles (fluorescence monitoring)	Wu, He et al. 2013
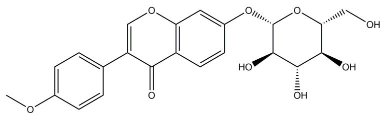	ononin	model membrane, *Escherichia coli*	cell membrane disruption	interaction of molecules with large unilamellar vesicles (fluorescence monitoring)	Wu, He et al. 2013
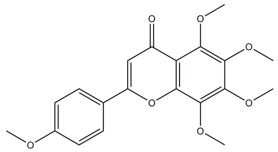	tangeritin	model membrane, *Escherichia coli, Pseudomonas fluorescens, Pseudomonas aeruginosa*	cell membrane disruption, DNA gyrase inhibition, reduced protein synthesis, inhibition of succinate dehydrogenase and malate dehydrogenase	efflux of cellular components, microscopy, DNA-gyrase supercoiling inhibition assay, interaction of molecules with large unilamellar vesicles (fluorescence monitoring)	Wu, He et al. 2013, Wu, Zang et al. 2013, Yao et al. 2011
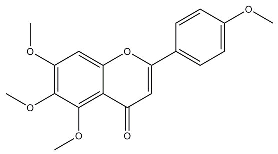	5,6,7,4'-tetramethoxy-flavone	model membrane, *Escherichia coli*	cell membrane disruption, DNA gyrase inhibition	DNA-gyrase supercoiling inhibition assay, interaction of molecules with large unilamellar vesicles (fluorescence monitoring)	Wu, He et al. 2013, Wu, Zang et al. 2013

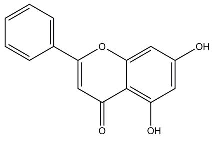	chrysin	model membrane, *Escherichia coli*	cell membrane disruption, DNA gyrase inhibition	DNA-gyrase supercoiling inhibition assay, interaction of molecules with large unilamellar vesicles (fluorescence monitoring)	Wu, He et al. 2013, Wu, Zang et al. 2013
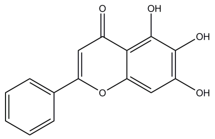	baicalein	model membrane, *Escherichia coli*	cell membrane disruption, DNA gyrase inhibition, Type III secretion inactivation	DNA-gyrase supercoiling inhibition assay, Type III secretion assay by monitoring fluorescence of Glu-CyFur dye, interaction of molecules with large unilamellar vesicles (fluorescence monitoring)	Wu, He et al. 2013, Wu, Zang et al. 2013, Tsou et al. 2016
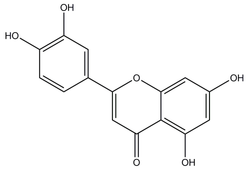	luteolin	model membrane, *Escherichia coli*	cell membrane disruption, DNA gyrase inhibition, Type III secretion inactivation, helicase inhibition (RepA)	DNA-gyrase supercoiling inhibition assay, Type III secretion assay by monitoring fluorescence of Glu-CyFur dye, ATPase activity assay with RepA, interaction of molecules with large unilamellar vesicles (fluorescence monitoring)	Wu, He et al. 2013, Wu, Zang et al. 2013, Xu et al. 2001

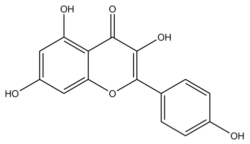	kaempferol	model membrane, *Escherichia coli*	cell membrane disruption, DNA gyrase inhibition, Type III secretion inactivation	DNA-gyrase supercoiling inhibition assay, Type III secretion assay by monitoring fluorescence of Glu-CyFur dye, interaction of molecules with large unilamellar vesicles (fluorescence monitoring)	Wu, He et al. 2013, Wu, Zang et al. 2013
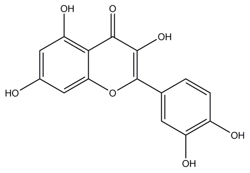	quercetin	model membrane, *Escherichia coli, Helicobacter pylori*	cell membrane disruption (membrane rigidification), DNA intercalation, DNA gyrase inhibition, Type III secretion inactivation, dehydratase inhibition (HpFabZ), protein kinase inhibition	DNA-gyrase supercoiling inhibition assay, type III secretion assay by monitoring fluorescence of Glu-CyFur dye, interaction of molecules with large unilamellar vesicles (fluorescence monitoring), kinase enzyme inhibition assays	Wu, He et al. 2013, Wu, Zang et al. 2013, Plaper et al. 2003, Tsou et al. 2016, Zhang et al. 2008, Shakya et al. 2011

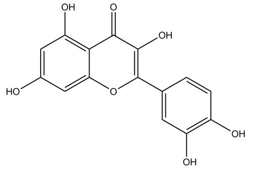	myricetin	model membrane, *Escherichia coli, Salmonella Typhimurium*	DNA gyrase inhibition, Type III secretion inactivation-ab, helicase inhibition (RepA)	DNA-gyrase supercoiling inhibition assay, Type III secretion assay by monitoring fluorescence of Glu-CyFur dye, ATPase activity assay with RepA, interaction of molecules with large unilamellar vesicles (fluorescence monitoring)	Wu, Zang et al. 2013, Tsou et al. 2016 Xu et al. 2001
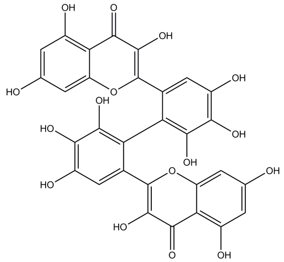	dimyricetin (oxidation product of myricetin)	*Escherichia coli*	helicase inhibition (RepA)	ATPase activity assay with RepA, interaction of molecules with large unilamellar vesicles (fluorescence monitoring)	Xu et al. 2001
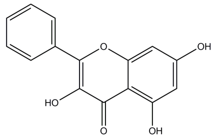	galangin	*Escherichia coli*	cell membrane disruption, DNA gyrase inhibition	efflux of potassium ions, DNA-gyrase supercoiling inhibition assay	Cushnie and Lamb 2004, Wu, Zang et al. 2013
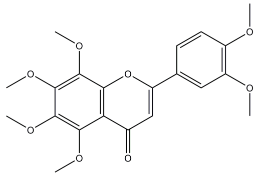	nobiletin	*Pseudomonas fluorescens, Pseudomonas aeruginosa, Escherichia coli*	cell membrane disruption, DNA gyrase inhibition, reduced protein synthesis, inhibition of succinate dehydrogenase and malate dehydrogenase	efflux of cellular components, microscopy, DNA-gyrase supercoiling inhibition assay	Yao et al. 2011, Wu, Zang et al. 2013
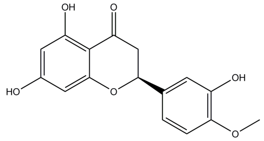	hesperetin	*Helicobacter pylori*	cell membrane disruption, urease inhibition	urease activity assay, microscopy	Moon et al. 2013

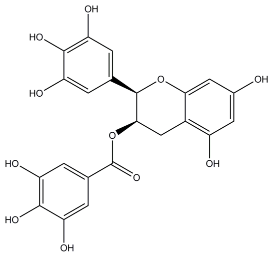	epigallocatechin gallate	model membranes, *MRSA, Bacillus subtilis, Escherichia coli*	cell membrane disruption (aggregation and inhibition of cell surface proteins, porin inhibition)	microscopy, purified cell wall components, phosphorus content, exogenous peptidoglycan added to activity assay, glass adherence, penicillin binding protein abundance/expression, Triton-X-autolysis assay, lysostaphin activity assay, collection of bacteriolytic enzymes and lipotechoic acid, mass spectrometry proteomics, interaction of molecules with multilamellar vesicles	Hashimoto et al. 1999, Kumazawa et al. 2004, Stapleton et al. 2007, Nakayama 2013, Nakayama 2015
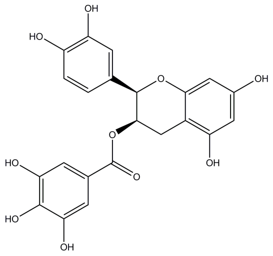	epicatechin gallate	model membranes, *Staphylococcus aureus*	cell membrane disruption (increased membrane autolysins, lipoteichoic acid release)	purified cell wall components, phosphorus content, exogenous peptidoglycan added to activity assay, glass adherence, penicillin binding protein abundance/expression, Triton-X-autolysis assay, lysostaphin activity assay, collection of bacteriolytic enzymes and lipotechoic acid, interaction of molecules with multilamellar vesicles	Hashimoto et al. 1999, Stapleton et al. 2007

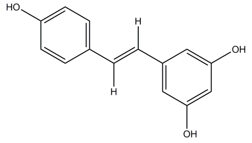	resveratrol	*Escherichia coli*	cell elongation (suppressed FtsZ expression), DNA fragmentation, increased SOS response	Z-ring inhibition assay, western blot, microscopy	Hwang et al. 2015
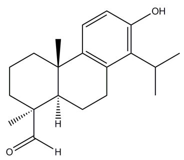	totaral	*Staphylococcus aureus*	reduced expression of enterotoxins, multi-drug efflux pump inhibitor	ethidium bromide accumulation/efflux assays, quantitative real time PCR	Shi et al. 2015, Smith et al. 2007
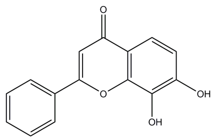	7,8-dihydroxy-flavone	*Salmonella Typhimurium*	Type III secretion inactivation	Type III secretion assay by monitoring fluorescence of Glu-CyFur dye	Tsou et al. 2016
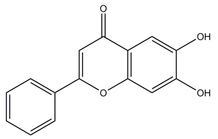	6,7-dihydroxy-flavone	*Salmonella Typhimurium*	Type III secretion inactivation	Type III secretion assay by monitoring fluorescence of Glu-CyFur dye	Tsou et al. 2016
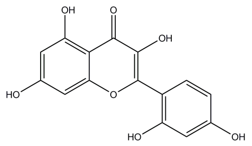	morin	*Salmonella Typhimurium*	Type III secretion inactivation, helicase inhibitor (RepA)	Type III secretion assay by monitoring fluorescence of Glu-CyFur dye, ATPase activity assay with RepA	Tsou et al. 2016, Xu et al. 2001
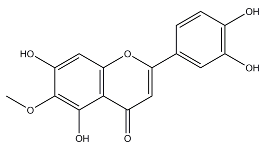	eupafolin	*Salmonella Typhimurium*	Type III secretion inactivation	Type III secretion assay by monitoring fluorescence of Glu-CyFur dye	Tsou et al. 2016
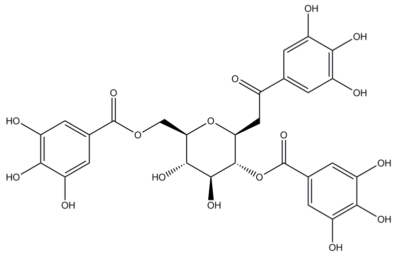	1,2,6-tri-O-galloyl-β-D-glucopyranose	*Escherichia coli*	multi-drug efflux pump inhibition	ethidium bromide accumulation/efflux assays	Bag and Chattopadhyay 2014

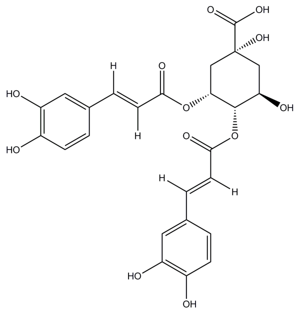	4,5-O-dicaffeoylquinic acid	no activity observed in tested range of mechanism study	multi-drug efflux pump inhibition	berberine accumulation/efflux assays	Fiamegos et al. 2011
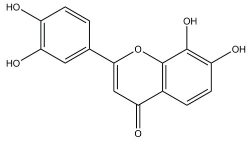	3’,4’,7,8 -tetrahydroxy-flavone	RecA purified from *Esherichia coli*	helicase inhibition (RepA)	ATPase activity assay with RepA	Xu et al. 2001
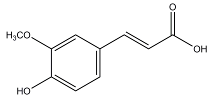	ellagic acid	*Salmonella Typhimurium*	DNA gyrase inhibition, not cell membrane	DNA-gyrase supercoiling inhibition assay, DNA-gyrase ”cleavable complex” assay	Ohemeng et al. 1993, Nohynek et al. 2006
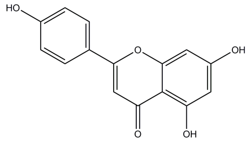	apigenin	*Helicobacter pylori*	dehydratase inhibition (HpFabZ), protein kinase inhibition	HpFabZ enzyme inhibition assay, interaction of molecules with large unilamellar vesicles (fluorescence monitoring), kinase enzyme inhibition assays	Zhang et al. 2008, Shakya et al. 2011
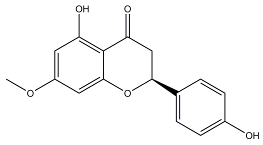	S-sakuranetin	*Helicobacter pylori*	dehydratase inhibition (HpFabZ)	HpFabZ enzyme inhibition assay, interaction of molecules with large unilamellar vesicles (fluorescence monitoring)	Zhang et al. 2008

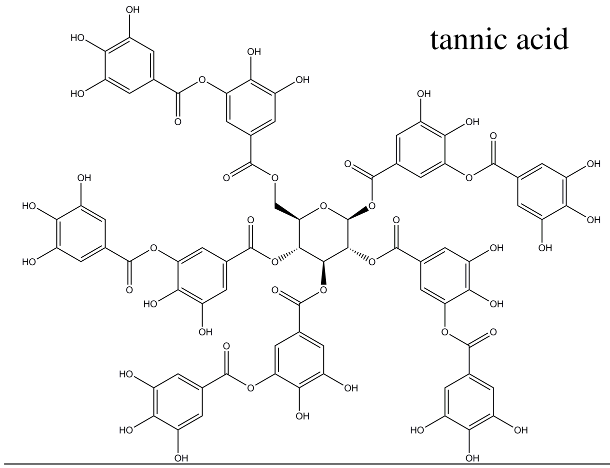	tannic acid	*Escherichia coli*	binds iron	iron binding assay, addition of exogenous iron	Chung et al. 1998

MRSA: methicillin resistant *Staphylococcus aureus*
HRGC: high resolution gas chromatography
